# Is German Medical Education Research on the rise? An analysis of publications from the years 2004 to 2013

**DOI:** 10.3205/zma000972

**Published:** 2015-08-17

**Authors:** Kristina Ackel-Eisnach, Patricia Raes, Lisa Hönikl, Daniel Bauer, Stefan Wagener, Andreas Möltner, Jana Jünger, Martin R. Fischer

**Affiliations:** 1 University of Koblenz-Landau, Faculty 5: Educational Sciences, Campus Landau, Department 1: Empirical Pedagogical Research, Landau, Germany; 2 Ludwig Maximilian University of Munich, Faculty of Medicine, Office of the Dean, Munich, Germany; 3 Clinic of the Ludwig Maximilian University of Munich, Institute for Medical Education, Munich, Germany; 4 Heidelberg University, Faculty of Medicine, Office of the Dean, Heidelberg, Germany; 5 Heidelberg University, Internal Medicine II, Heidelberg, Germany; 6 Heidelberg University, Centre of Competence for Medical Testing Baden-Württemberg - KomP Med, Heidelberg, Germany

**Keywords:** medical education research, literature analysis, content analysis, international publication

## Abstract

**Objectives: **The point of departure for the present work is the observation that, in comparison with Anglo-American countries or the Netherlands, Germany was responsible for only a marginal number of international publications in the field of medical education research before 2004. Recent years, however, have seen an increase in the importance of medical education research in Germany. The objective of this article is to evaluate the extent to which this trend can be substantiated by increased German publishing activity since the year 2004 in international, English-language journals in the subject area of “medical education research”.

**Methods: **In the framework of a literature and content analysis, German-author articles from the years 2004 to 2013 in six international, English-language medical education research journals were evaluated. In order to obtain an overview of German research activity in this area, all project and original works with German first and last authors were identified and subjected to a more in-depth content analysis.

**Results:** In total, 10,055 articles were examined. The evaluation shows that between the years 2004 and 2013 179 articles (of which 145 are project or original works) by German authors were published in the journals examined. Fluctuations over the course of time were evidenced. The project and original works are primarily cross-sectional studies (27.8%) and randomised control studies (25.6%) on the subject of “teaching and learning methods” (43.6%).

**Conclusions:** In comparison with the years 2004-2008, a significant rise can be seen in the number of publications by German education researchers in international journals since the year 2009.

## Zusammenfassung

**Zielsetzung: **Ausgangspunkt des vorliegenden Artikels ist die Feststellung, dass es aus Deutschland im Vergleich zu den angloamerikanischen Ländern oder den Niederlanden vor 2004 kaum internationale Publikationen im Bereich der medizinischen Ausbildungsforschung gab. In den letzten Jahren wurde jedoch eine steigende Bedeutung der medizinischen Ausbildungsforschung im deutschen Raum deutlich. Zielsetzung dieses Beitrags ist es zu prüfen, inwieweit sich in internationalen, englischsprachigen Fachzeitschriften im Themenfeld „Medizinische Ausbildungsforschung“ seit 2004 eine solche Entwicklung durch eine gesteigerte Publikationsaktivität belegen lässt.

**Methodik:** Im Rahmen einer Literaturauswertung und Inhaltsanalyse wurden Artikel deutscher Autoren aus den Jahren 2004 bis 2013 in sechs internationalen englischsprachigen Fachzeitschriften der medizinischen Ausbildungsforschung analysiert. Um einen Überblick über die deutschen Forschungsaktivitäten in diesem Bereich zu bekommen, wurden alle Projekt- und Originalarbeiten deutscher Erst- und Letztautoren identifiziert und einer tiefergehenden inhaltlichen Analyse unterzogen.

**Ergebnisse**: Insgesamt wurden 10.055 Artikel untersucht. Die Auswertung zeigt, dass zwischen 2004 und 2013 179 Artikel – hiervon 145 Projekt- und Originalarbeiten – deutscher Autoren in den betrachteten Zeitschriften publiziert wurden. Es zeigen sich Schwankungen im Zeitverlauf. Bei den Projekt- und Originalarbeiten handelt sich vor allem um Querschnitt- (27,8%) und randomisierte Kontrollstudien (25,6%) zum Thema „Lehr- und Lernmethoden“ (43,6%).

**Schlussfolgerung: **Seit 2009 zeigt sich ein deutlicher Anstieg der Publikationszahlen deutscher Ausbildungsforscher in internationalen Fachzeitschriften im Vergleich zu den Jahren 2004-2008.

## Notes

The authors (Kristina Ackel-Eisnach, Patricia Raes, Jana Jünger, Martin R. Fischer) contributed in equal parts to the publication (shared first and last authorship).

## 1. Introduction

As early as 2004, the German Council of Science and Humanities indicated that inadequate attention was being paid to the teaching of medicine in relation to the importance placed on medical research and patient care. In the scope of its recommendations, the Council additionally states that educational research was only sporadically established and professionalised at the time [1]. In recent years, however, a growing appreciation of and increasing interest in medical education research have been observed [2], [3]. The German Medical Faculty Association (MFT) and the German Medical Association (GMA) jointly advocate the establishment of educational research as an engine for medical education in Germany [4]. This trend is illustrated by, among other things, the positive trend in numbers of visitors accessing articles from the open-access medical education journal GMS - German Medical Science (GMS Z Med Ausbild), averaging 5,638 per month in 2013 (see Table 1 (Tab. 1)).

The journal serves as the publication organ of the GMA and has focused thus far on medical education research projects in German-speaking Europe [4]. This field enjoys further esteem through the establishment of the “Master of Medical Education (MME)” programmes in Germany and Switzerland [5].

In addition, the significance of educational research in German-speaking Europe is clearly evidenced by the increase in the number of GMA members. Membership rose from 200 in the year 2004 to over 1,000 in 2014. In Heidelberg in the year 2009, the initiation of the biennial “Research in Medical Education” conferences (RIME) by the MME programme of the German Medical Faculty Association (MFT) further bolstered the importance of German-speaking Europe in international medical education research. 

International information exchange represents an essential aspect of scientific research. This comprises the publication of research projects and their results in international, English-language journals, on the one hand, and the presentation of research projects to an international audience, e.g. at conferences, on the other. Raes et al. were able to show that although the active participation of German-speaking educational researchers at the conferences of the Association for Medical Education in Europe (AMEE) fluctuated in the evaluated period of 2005 to 2013, their number has risen continually since the year 2010 [3].

Numerous international journals exist in the field of “Medical Education”. Unlike publications in the life sciences, these journals contain only marginal numbers of articles from Germany up until the year 2004 [4], [6]. The objective of the present article is to inventory the publications of medical education researchers from Germany in international, English-language journals during the past ten years (2004 - 2013). The article is purely descriptive in design and attempts to elaborate the following aspects:

- How many articles – particularly project and original works – by educational researchers from Germany were published in the international, English-language journals in the course of this time period?
- Can the articles be classified according to various criteria?
- What are the subjects and main topics of the articles?

## 2. Methods

In the empirical analysis of the international visibility of German educational researchers, the present article draws from the following selection of international, English-language journals: *Medical Education* (JIF 2013=3.617), *Medical Teacher* (JIF 2013=2.045), *Academic Medicine* (JIF 2013=3.468), *Advances in Health Sciences Education* (JIF 2013=2.705), *Teaching and Learning in Medicine* (JIF 2013=1.173) and *BMC Medical Education* (JIF 2013=1.41). In the view of the authors of this article, these are the particularly visible and relevant international journals for the year 2004. All of these journals have an editorial board and are subjected to a peer review process. *Medical Education, Medical Teacher* and *Academic Medicine* are three of the long-standing journals in the area of medical education, further training and advanced studies that are held in high esteem among experts in the field. The first edition of *Medical Education* was published in 1966 under the title “British Journal of Medical Education”, while Medical Teacher first appeared in 1979. The journal *Academic Medicine* was first published in 1974 under the name “Journal of Medical Education”. The journals *Teaching and Learning in Medicine* and *Advances in Health Sciences Education* have been in publication since 1989 and 1996 respectively. *BMC Medical Education* is an open-access journal that was first published in 2001.

The specified journals served as units of analysis in the evaluation. The relevant observation period for the articles ranges from 1 January 2004 to 31 December 2013. All articles published within the observation period were examined. Thus, 563 individual issues and 10,055 articles in total were included in the analysis. Each individual journal article served respectively as the unit of observation in the present evaluation. All printed contributions as well as e-publications were classified as articles. All articles published in the specified journals were identified as relevant when at least one author’s country of work was Germany.

**Figure 1:**
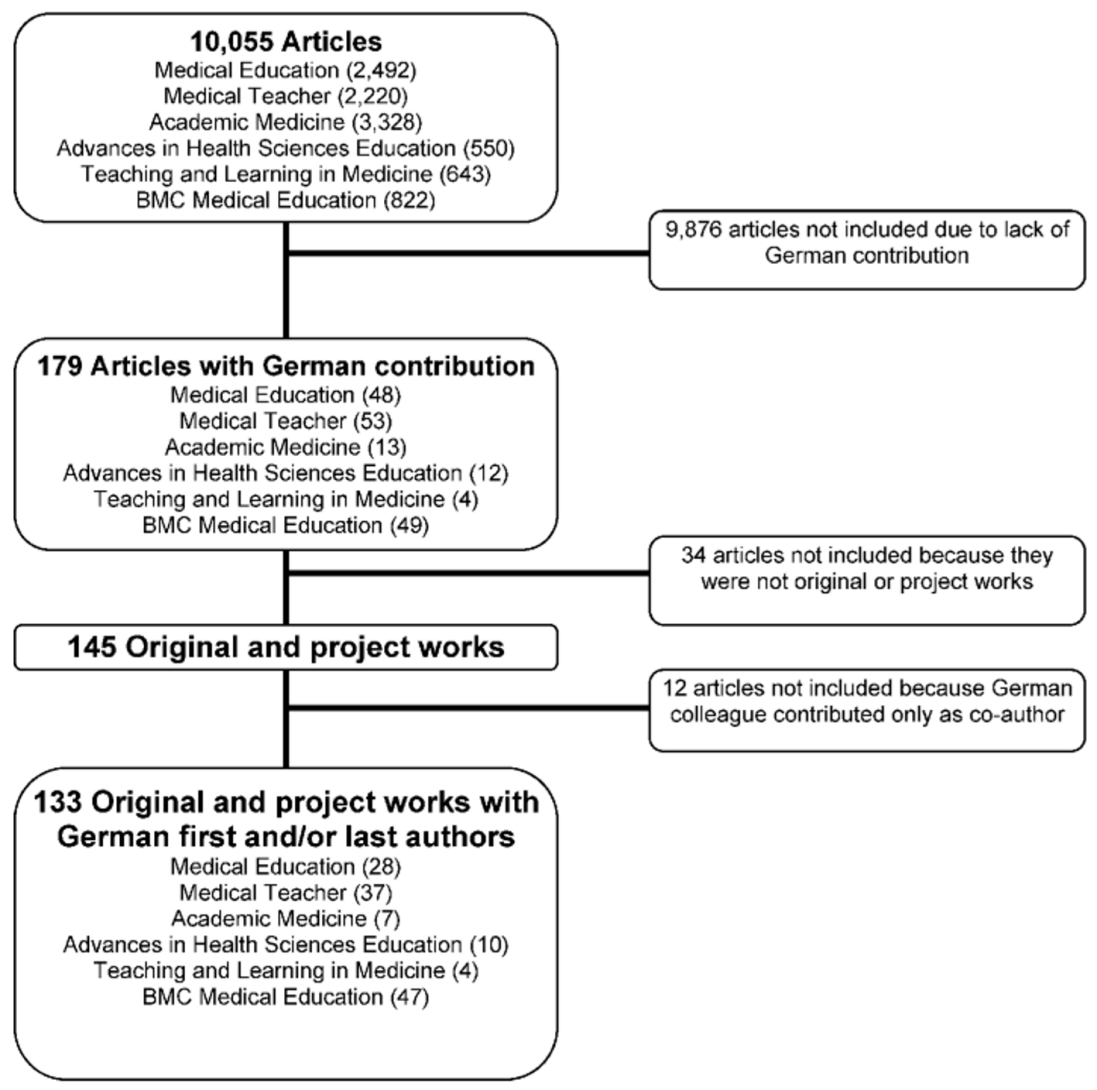
flowchart describing the article selection from six designated international, English-language journals on medical education

Following the selection of relevant articles, each article was classified by type. In this way, all original and project works were identified and subjected to further examination in the form of content analysis (frequency analysis – see Figure 1 (Fig. 1)). 

Because they do not offer complete representations of new scientific findings in the field of medical education research in comparison to that of original and project works, articles falling into the category “further contribution” (e.g. editorial, letter to the editor, review) were not considered. The subsequent analysis of the identified project and original works involved encoding them according to research design, data type, object of study and research topic. Classification of research topics was oriented on that of Raes et al [3]. Furthermore, classification by *“description”, “justification”* and *“clarification”* was effected following the methods of Cook et al [7], [8]. *“Description studies”* are at the lowest level of investigation and usually contain descriptions of the interventions introduced or curricular processes. Results data can but may not necessarily be part of the study. Based on group comparison,* “justification studies”* go one step further and attempt to prove the effectiveness of an intervention. By use of model assumptions, *“clarification studies”* additionally attempt to clarify why or how an intervention works. 

## 3. Results

From 2004 to 2013, 179 articles by German authors were published in the examined international, English-language journals. This equates to 1.8% of the total number of articles published in this time period. A marked difference was noted at the level of the individual journal (see Figure 1 (Fig. 1)).

### 3.1 Number of project and original works

**Figure 2:**
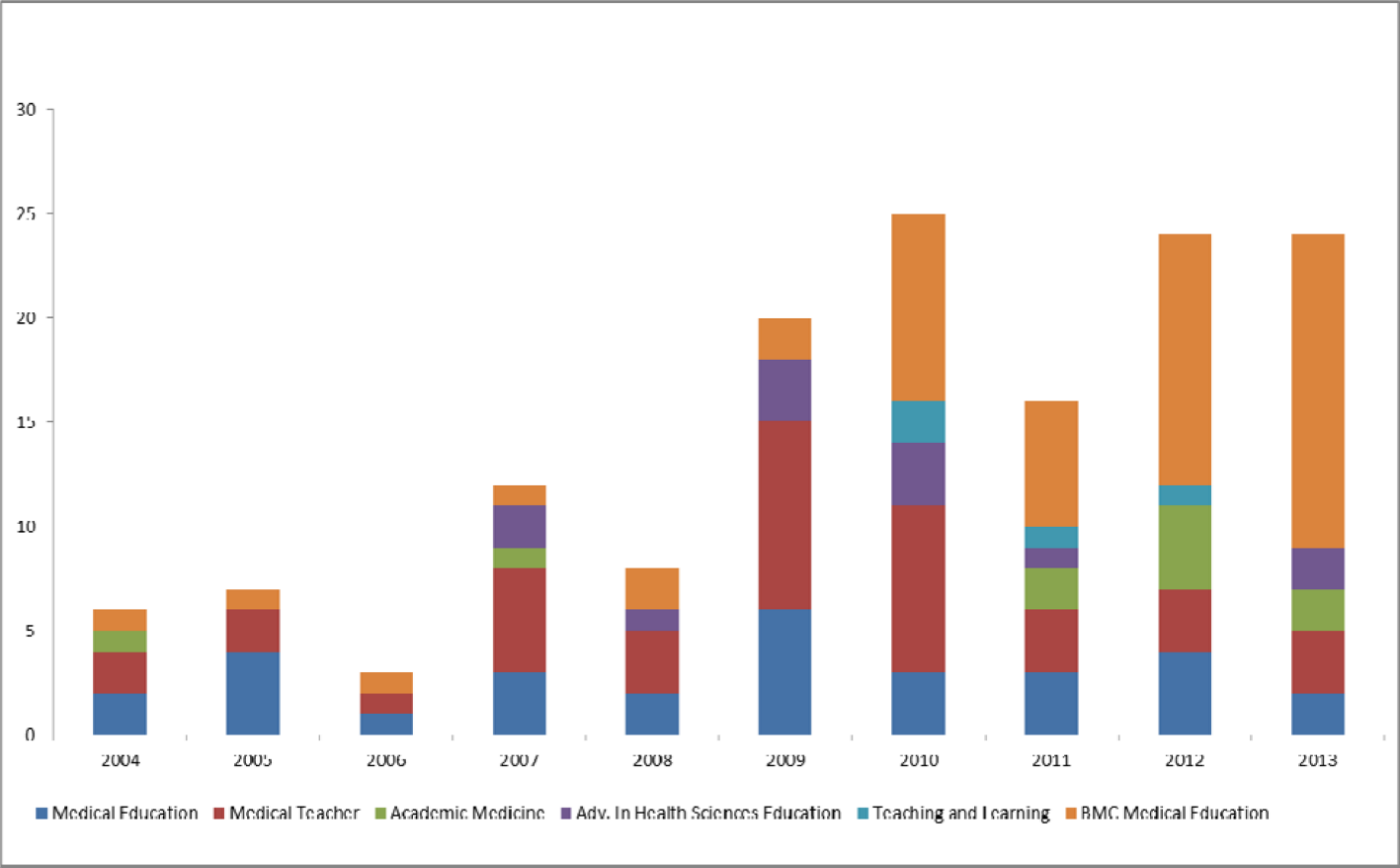
number of project and original works with German participation by year and journal (n=145).

Project and original works represent a large portion of the publications by German authors in international, English-language journals. In total, 145 project and original works with German contribution (see Figure 2 (Fig. 2)) or 133 project and original works with German first and/or last authorship were published. Thus, of the published articles with German contribution, 81.0% were project or original works.

**Figure 3:**
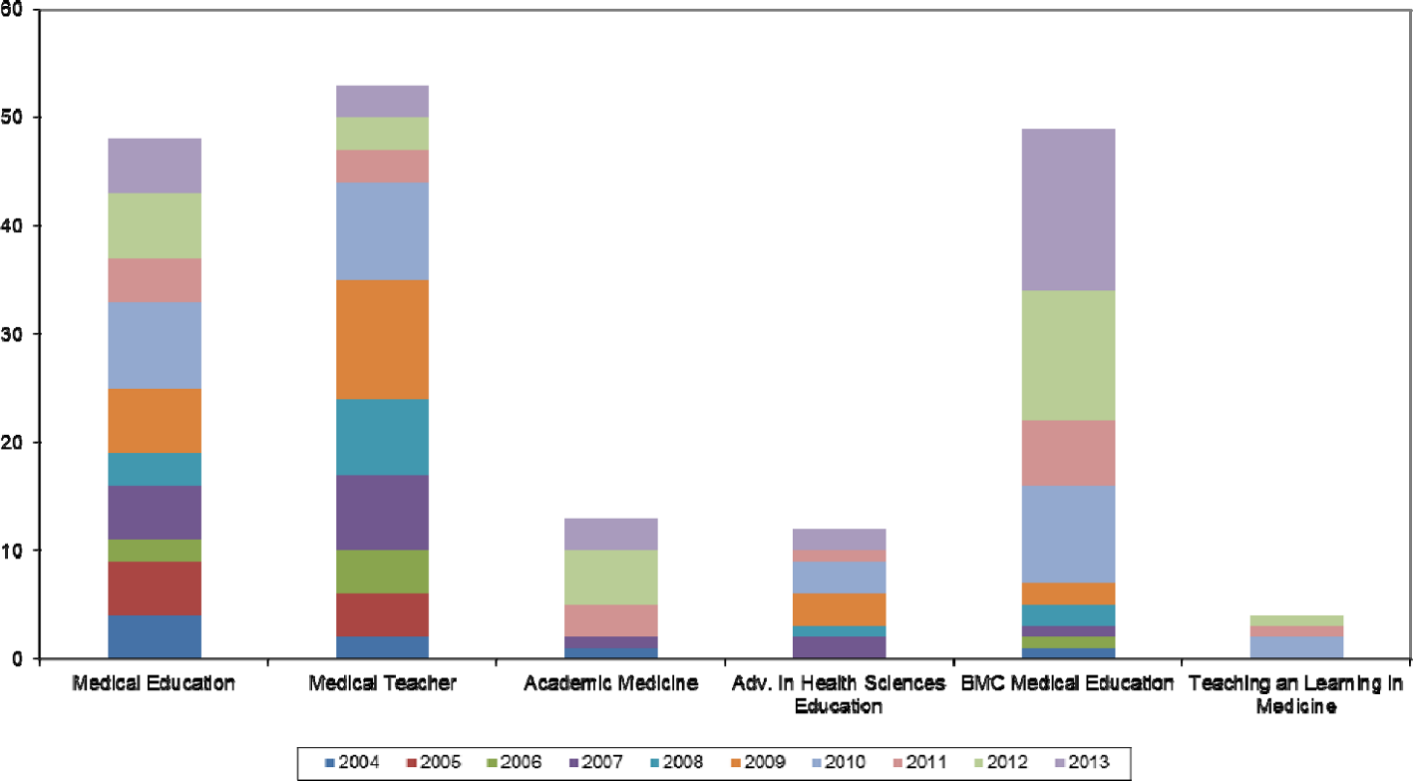
Number of publications with German participation by year and journal (n=179)

Figure 3 (Fig. 3) shows the total number of articles with German contribution ordered by journal. German authors are particularly prevalent in *Medical Education, Medical Teacher* and *BMC Medical Education*. The journal *BMC Medical Education* shows a considerable increase in publications with German contribution in recent years. 

#### 3.2 Methodological and thematic categorisation of project and original works

The project and original works with German first or last authorship primarily involved studies with ex post facto design, pre-experimental and experimental design. Table 2 (Tab. 2) and table 3 (Tab. 3) specifies the categorisation by study design. For the most part, quantitative data was gathered (see table 2 (Tab. 2)). In most cases, students in the clinical phase of their programme are the object of study. Central thematic emphasis is on teaching and learning methods, changes in curriculum and examination procedures. In the original and project works, *“clarification studies”* are 42.1% more common than *“justification studies”* (38.3%).

#### 3.3 Original and project works according to faculty

**Figure 4:**
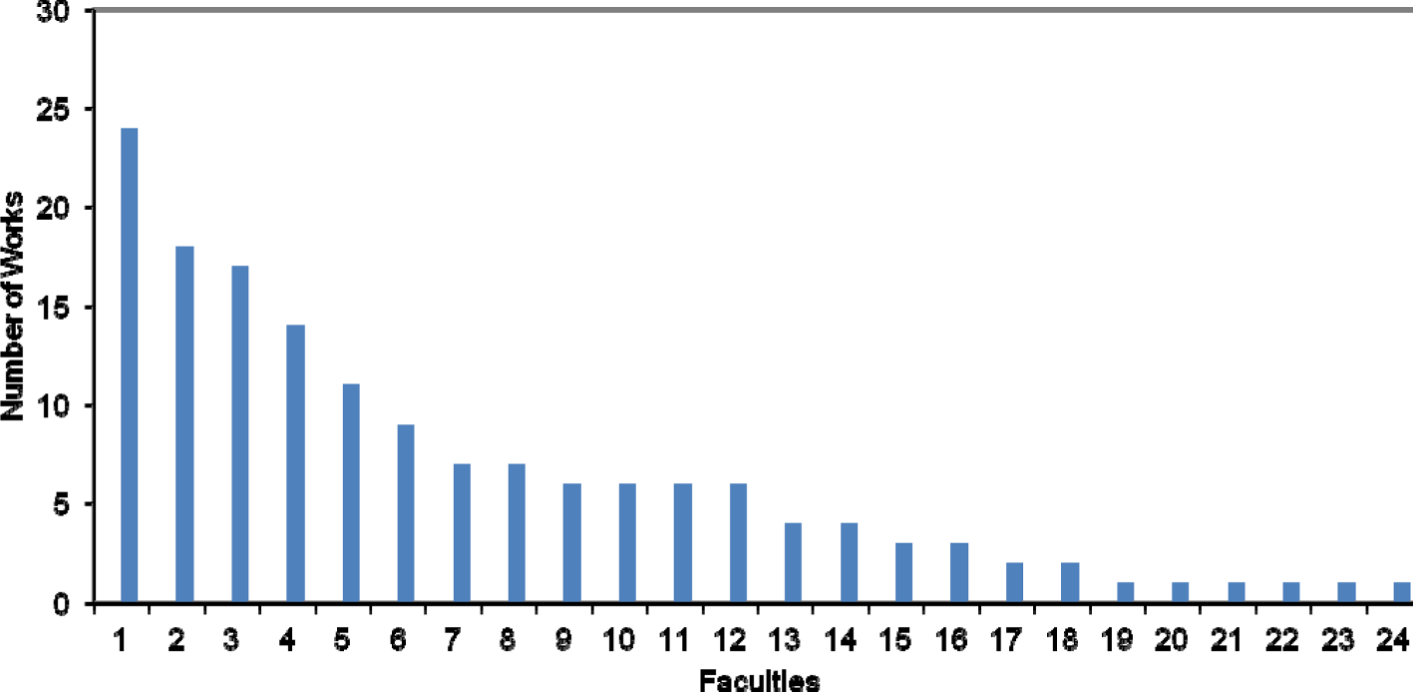
Number of published original and project works in the observation period sorted by faculties in Germany with at least one publication (only articles with German first or last authors). When first and last author belonged to the same faculty, the article was only counted once. When first and last author belonged to different faculties, the article was counted twice. When first and last authorship were shared, only the faculty of the first-named author was considered.

The individual medical schools in Germany participate in international publications to varying degrees (see figure 4 (Fig. 4)). Some of the faculties publish regularly in the six journals examined, while others were scarcely represented or entirely absent in the period examined. In descending order, the five German medical schools with more than ten internationally published original and project works are: Heidelberg University, Ludwig Maximilians University Munich, Berlin (Freie Universität Berlin and the Humboldt University of Berlin), the University of Hamburg and the University of Göttingen.

## 4. Discussion

Observations revealing that representatives of German medical faculties only marginally addressed medical education research in the past served as the point of departure for the present research. Simultaneously, a certain lag in the international visibility of German educational researchers was detected. Doja et al. were able to show that Canada, the Netherlands, New Zealand, Great Britain and the USA were the countries with the highest relative productivity in the field of medical education research [9], while Germany only ranked eleventh in the study. Consequently, the objective of the present article was, in the scope of a content analysis of selected international, English-language journals on “medical education research”, to investigate to what extent international publication activity by German researchers in the time since 2004 is rising.

Overall, no distinct trend was detectable from the number of international publications with German authorship. Although the number of German-author project and original works published in international, English-speaking journals was clearly greater between 2009 and 2013 than in the years 2004 to 2008, fluctuations over the course of time suggest the need of further observation and analysis for potential trend analyses. Individually seen, the only journal that exhibits a positive trend regarding German publication in the observation period is *BMC Medical Education.*

Pursuant to the investigations of various authors, studies in the area of medical education research are often based on inadequate scientific approaches and research designs [8], [10], [11], [12]. The results of the present study show, however, show that the original and project works by German authors are fortunately *“clarification”* and *“justification”* studies. In comparison, contributions by German-speaking researchers at AMEE conferences are seldom clarification studies. Between 2005 and 2013, German-speaking researchers chiefly presented descriptive studies at the conferences [3]. The most frequently applied research designs found during the present analysis were of an experimental nature (pre-experimental research design and randomised control studies). In articles analysing research methods in medical education research, Baernstein et al. came to similar conclusions [13]. They were able to demonstrate that 25% of the studies between 2004 and 2007 examined randomised control groups. As shown in the present work, while the percentage of German-language contributions in the total number of presentations at AMEE conferences between 2005 and 2013 is 5.8%, the percentage of international journal articles by German-language authors in the six examined journals is, at 1.8%, significantly lower. It must be taken into account that Raes et al. included Austria and Switzerland in their analysis but not in the present work. It is possible that a majority of the descriptive studies are published less frequently in the examined international journals [3]. The descriptive contributions may be more likely to appear in German-language journals. Comparative empirical data on this is, however, not available. 

The most common research topics, methods and objects of investigation in studies by German-speaking researchers presented at the AMEE conferences match those of the current analysis.

Medical schools in Germany participate in medical education research to differing degrees, as Raes et al. have already demonstrated [3]. The present analysis confirms this. The faculties in Heidelberg, Munich and Berlin are among the five most frequently published German faculties in the field of medical education research. Thus, it appears that particular faculties are more successful at publishing internationally. This result may reflect differing developments among German medical schools [3]. Some German faculties instigated teaching reforms and the implementation medical education research in their schools previous to any official dictate. Other German medical schools assigned medical education more significance only in the course of the implementation of the revised German Medical Licensure Regulation (Approbationsordnung – revision effective from 1 October 2003). In addition, experts who are capable of meeting the demanding requirements of medical education research are lacking in the faculties [6].

If we see publishing in international journals not only as a means of distinguishing oneself but also as an opportunity to actively influence the selection, content and methods of articles appearing in the journals, then the relatively low international publishing activity in the field of medical education research reveals a further deficit. Growing multi-national influence on the examined journals inherently entails their internationalisation [14]. The trend of increase in German contributions to international journals on educational research shows a first form of direct influence that can be made on the content of a journal. Furthermore, authors who regularly publish in international journals are highly likely to become reviewers for those journals, affording yet another form of influence over the content of the journals. Editing allows for a third form of influence over journal content. Currently, Medical Education and Medical Teacher each have a colleague from a German institution working on their editorial boards. Five German colleagues are active as editors for* BMC Medical Education*. These three journals published a notably higher number of articles by German researchers in the observation period. The high number of German editors could also account for the increase in German-author publications in recent years. The other three journals do not currently have any German representatives on their editorial boards. 

Despite application of the aforementioned criteria, the selection of journals scrutinised doubtlessly represents a limitation to the validity of the analysis results. This is due in part to the fact that other international journals on the topic of “medical education research” were not considered in the analysis. In addition, important contributions to medical education research also appear in other medical specialist journals (e.g. Anatomical Sciences Education) or in non-medical, educational sciences journals, such as Learning and Instruction. This is evidenced by observations of international publications submitted within the framework of the “Master of Medical Education” (MME) programme of the Medical Faculty Association (MFT) at the University of Heidelberg. From 2006 to 2012, a total of 22 international publications by graduates of the programme appeared, of which only eight were published in the journals analysed in the present research. The other 14 publications appeared, for the most part, in journals with medical-clinical contents (e.g. Resuscitation) or, to a lesser degree, in the *European Journal of Dental Education*. The fact that the majority of those completing this postgraduate course of studies work in clinical fields appears to influence the choice of journal for publication. 

Furthermore, it must be noted that, although the ten-year time period considered during the present evaluation is relatively long, articles published before 2004 and after 2013 were not taken into account in the analysis. Further to the investigation of the prevalence of articles by German medical education researchers in international journals, an additional study on topics and research questions in international comparison would be enlightening. Establishing which German medical education researchers are most frequently cited could also be of interest. An investigation of whether language barriers, method deficiencies or other factors are the main reason for the relatively low visibility of medical education research in German-speaking Europe would also prove insightful. 

## 5. Concluding remark

As in every academic discipline, medical education needs solid standards and qualified experts. This area has seen gradual structural improvement in Germany. The presence of articles by German educational researchers reflects this development. In comparison with the amount of German publications in international life science journals, the progress made in German educational research in recent years is only marginal, and the field is doubtlessly worthy of support and capable of development. Despite these critical remarks, the authors of this article hope that the present work demonstrates that international essays in the field of medical education research are of significant value and have increased in number over the last five years, when compared to the years 2004 through 2008. In light of their significance, it appears to be necessary to pool the diverse insights and approaches in this field and to strengthen the contact between national and international researchers and users.

## 6. Competing interests

The authors declare that they have no competing interests.

## Figures and Tables

**Table 1:**
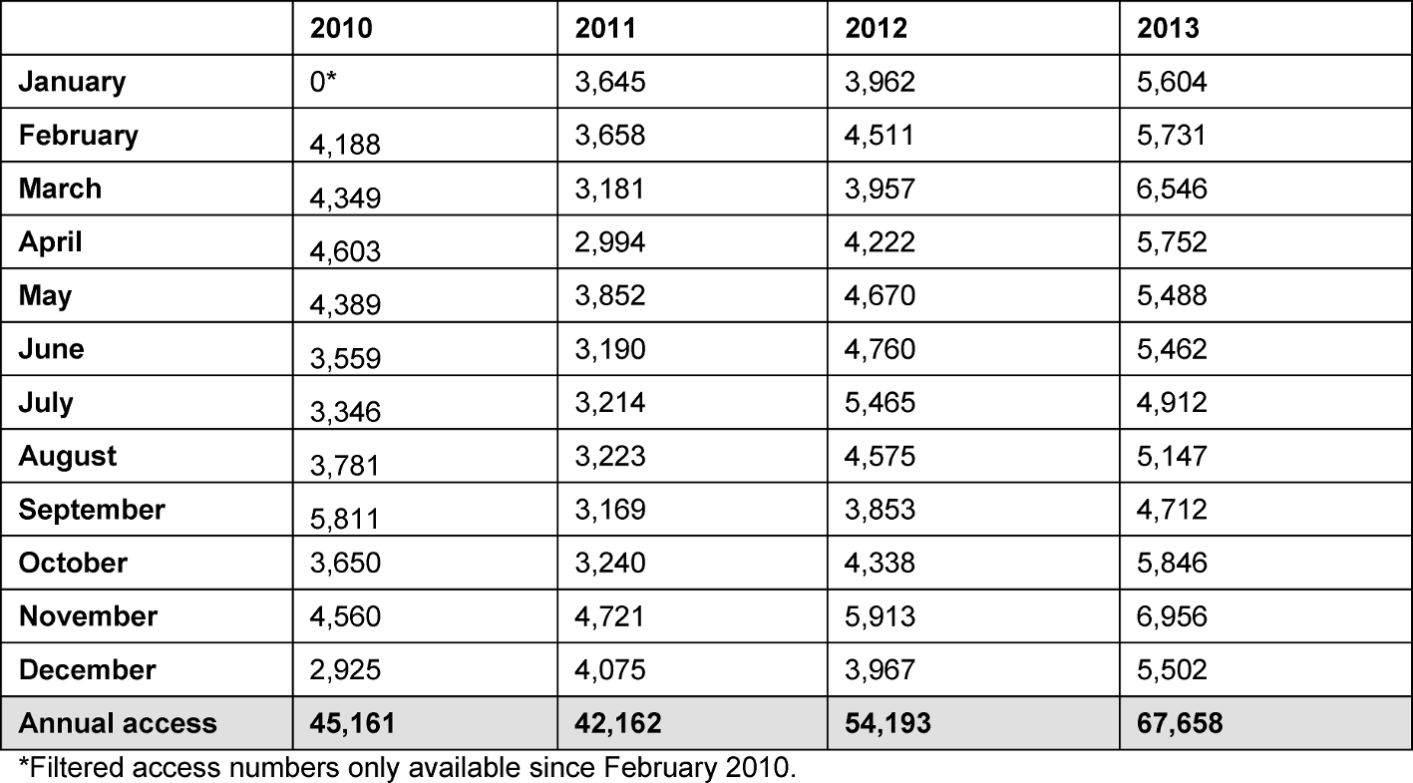
monthly / yearly user access via the open-access site of the medical education journal GMS between 2010 and 2013

**Table 2:**
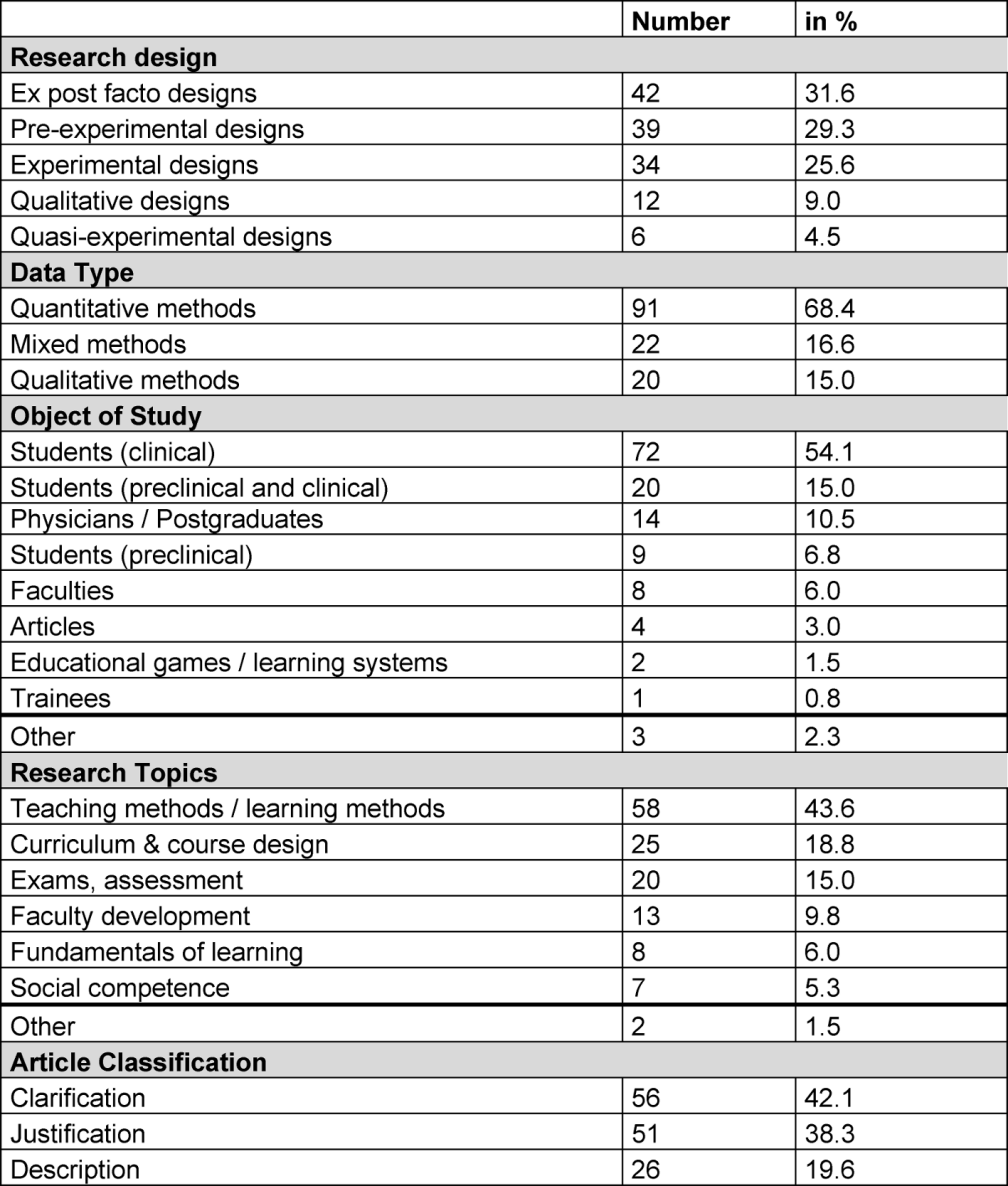
categorisation of project and original works with German first or last authors using various criteria (n=133)

**Table 3:**
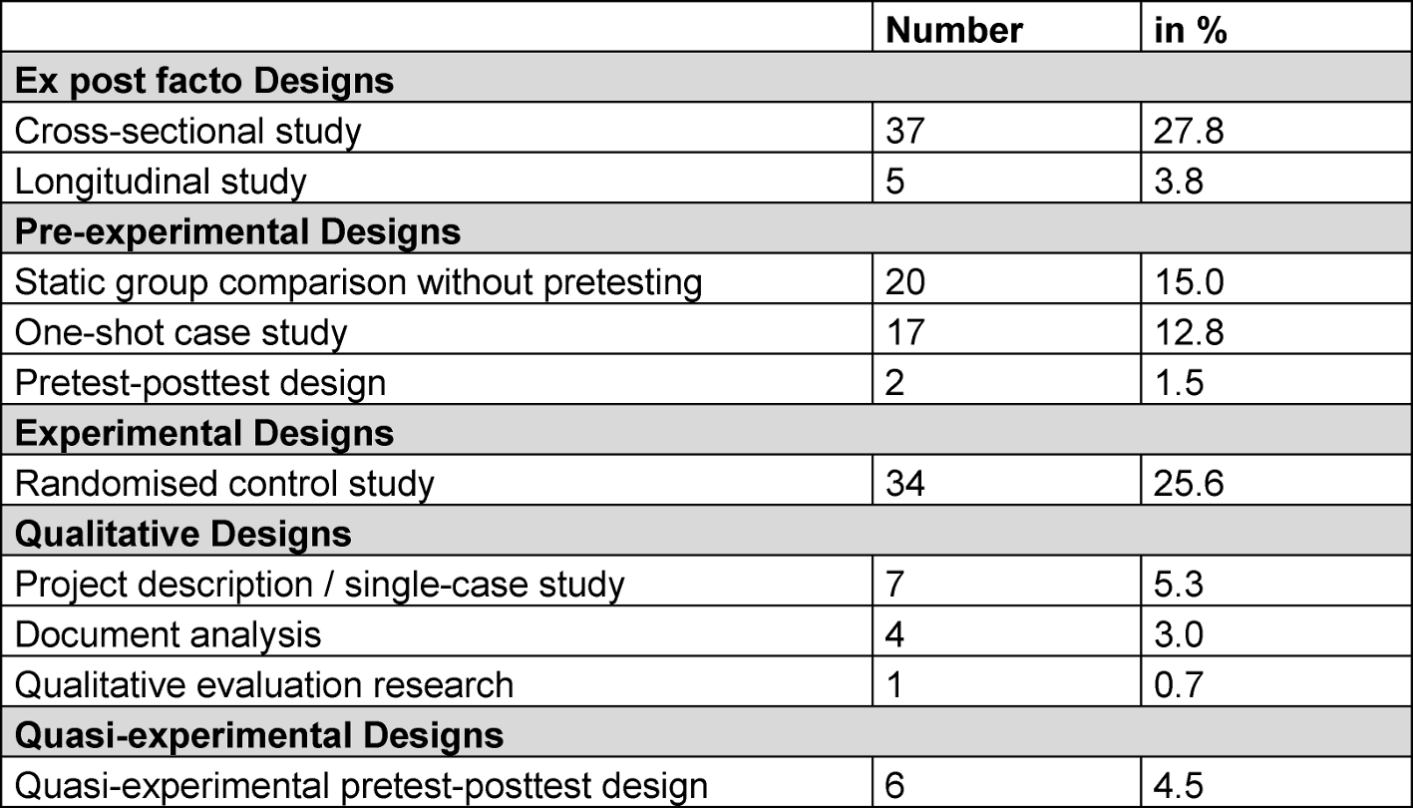
categorisation by research design of project and original works with German first or last authors (n=133)
